# Nutritional Value of Brewer’s Spent Grain and Consumer Acceptance of Its Value-Added Food Products

**DOI:** 10.3390/foods14162900

**Published:** 2025-08-21

**Authors:** Victoria Eche, C. U. Emenike, H. P. Vasantha Rupasinghe

**Affiliations:** 1 Department of Plant, Food, and Environmental Science, Faculty of Agriculture, Dalhousie University, Truro, NS B2N 5E3, Canadachijioke.emenike@dal.ca (C.U.E.); 2 Department of Natural and Applied Sciences, Hezekiah University, Umudi 471125, Nigeria

**Keywords:** Brewer’s spent grain, food, health, consumer, nutrients, valorization

## Abstract

Brewer’s spent grain (BSG), a byproduct of the brewing process, offers a sustainable alternative applicable to human nutrition. The nutritional composition, health advantages, and value-added uses of BSG in diverse food items, including snacks, bread, cookies, and pasta, are examined in this review. Furthermore, consumer acceptance and organoleptic attributes, including texture, taste and appearance, are discussed. BSG is composed of 60% carbohydrates (of which 50% dietary fiber), 10% lipids, and 30% proteins. BSG is also high in minerals such as calcium and phosphorous and bioactive polyphenols such as catechin, *p*-coumaric, and ferulic acid. BSG holds significant opportunities to be utilized in enhanced food production, biofuel generation, and other industrial applications. The reported therapeutic effects of BSG include anticarcinogenic, antiatherogenic and oxidative stress reduction. Based on sensory evaluations, the maximum amount of BSG that can be added to food products to maintain consumer acceptance is 15%. There is a need to convince manufacturers and consumers of the potential of incorporating BSG into food products, the health benefits of this, and the sustainability advantages of the use of BSG. The integration of BSG into food systems will contribute to food waste minimization and the promotion of the circular economy.

## 1. Introduction

Plant-based foods derived from a variety of vegetables, fruits, cereals, nuts, seeds, and pulses are essential for human nutrition and overall health. With increasing emphasis on sustainability, the efficient utilization of plant-based resources becomes critical. Agro-industrial waste is a major concern due to the rising global demand for food. The global requirement for food fuels the need for the identification of cost-effective raw materials for sustainable production while meeting dietary requirements [1]. The disposal of byproducts in the food and beverage sector creates cost and environmental challenges, necessitating innovative solutions.

Brewer’s spent grain (BSG), a major residue generated during beer manufacturing, is primarily derived from malted barley, and it may additionally consist of maize, oats, and wheat. Approximately 20 kg of BSG is produced for every hectoliter of beer brewed [2,3]. North and South America collectively produce approximately 12.3 million tonnes of BSG annually [4]. Barley undergoes soaking, germination, and drying during brewing to extract fermentable sugars, leaving behind nutrient-rich BSG [5].

BSG constitutes approximately 85% of brewing waste, with spent hops (1–2%) and spent yeast (13–15%) forming the remainder. Traditionally utilized as livestock feed, BSG is currently being repurposed for diverse uses, including biofuel generation and applications in building materials, packaging, and food production [6,7]. The inclusion of valuable nutrients such as protein, fiber, and micronutrients has increased interest in its food applications [8,9]. BSG can be used as a value-added ingredient in baking, snack foods, and even juices, enhancing nutritional profiles and adding unique flavors. Moreover, repurposing BSG also contributes to the circular economy by transforming what would be considered waste into valuable food resources [3]. There has been growing consumer interest in the role of plant-based proteins emerging as an alternative to animal proteins [10], making BSG a potential dietary source of protein. The recovery of nutrients from brewing wastes may thus represent an innovative and highly valuable source for the food industry, in addition to revenue recovery due to waste diversion [9]. The valorization of BSG aligns with the United Nations sustainability goals, promoting the best use of renewable resources and reducing environmental impact [11]. Disseminating knowledge on the composition, physicochemical properties, potential valorization pathways, and research significance of brewing industry byproducts represents an effective strategy for enhancing their economic and societal value.

Despite its potential, challenges such as economic feasibility and processing hinder BSG’s widespread adoption. Processing and quality control costs may offset its affordability as a raw material. Additionally, significant research gaps remain in the characterization and mechanistic understanding of the physicochemical, rheological, and structural properties across diverse food matrices. This review examines the behavior of BSG in baked goods, beverages, and other food products while analyzing consumer perceptions and sensory attributes.

### Methodology

The review was conducted by using databases including SCOPUS, Google, Scholar, Web of Science, and ResearchGate for studies published between 2013 and 2024. Relevant publications before 2013 were also considered. Inclusion criteria included studies addressing the nutrient composition of BSG and its benefits, and its food applications. Search terms included BSG, “chemical composition of BSG”, “proximate composition of some cereals and legumes/pulses”, “BSG uses in food application”, “health benefits of BSG”, and “BSG use in baked products, pasta, yogurt and juices”. The excluded studies focused on non-food applications such as biofuels and animals. The quality and number of participants used for sensory assessment studies were also considered in selecting articles.

## 2. Nutritional Profile of BSG

BSG is rich in nutrients, making it useful in both food and nutraceutical applications (Table 1). Its primary components include the husk, pericarp, and seed layers of barley grains, along with residual starchy endosperm. Variability in BSG composition arises from differences in barley variety, hop type, harvest time, malting and mashing procedures, and the presence of brewing [6]. Regardless of these variations, BSG is characterized as a lignocellulosic that is high in moisture (80%), and significant amounts of cellulose, hemicellulose, lignin, fiber, and protein [10].

BSG encompasses various components such as carbohydrates, hemicellulose, fiber, ash, protein, amino acids, lignin, minerals, vitamins, sugar, and polyphenols (Table 1). Hemicellulose fraction in BSG of 15–30% is predominantly composed of arabinoxylan, a functional dietary fiber that contributes to improved antioxidant effects, prebiotic support, and glycemic regulation [6,11,39]. Although BSG is typically categorized as a lignocellulosic byproduct, it also has a substantial protein fraction, up to 30%. This makes it compositionally similar to barley, its primary source [13,40]. Hordeins represent the main protein group in BSG, notably B-hordeins (30–50 kDa) and C-hordeins (55–80 kDa), accompanied by peptides, essential (leucine, isoleucine and valine up to 10%), and non-essential amino acids (majorly glycine and glutamic acid of about 15%); most of them are generated by enzymatic hydrolysis throughout the brewing process [10,12,31,41].

On a dry basis, about 60% of BSG comprising undigested non-carbohydrate polysaccharides and digested carbohydrates, which are normally considered fibers. Also on a dry basis, sugars like maltose, arabinose, glucose and xylose are present in BSG, contributing not more than 25% of the entire mass. Lipids are present in BSG in small quantities, with triglycerides comprising the major portion of the lipid fraction (approximately 67% of total extract), followed by approximately 18% fatty acid and lower quantities of monoglycerides and diglycerides of 1.6 and 7.7%, respectively [1,27,28,42]. Calcium, magnesium, phosphorus, and sodium, which are considered macro minerals, are also present in BSG. Furthermore, BSG is valued for its high content of phenolic compounds, of which ferulic acid (a hydroxycinnamic acid) is the most abundant, with a concentration of almost 1144 μg/g, followed by *p*-coumaric acid, which has a concentration of 453 μg/g. Hydroxybenzoic acid and hydroxycinnamic acids are secondary plant metabolites extensively found in plant foods. This is because since BSG is largely made up of husk, pericarp, and seed coat, which are rich in cell wall material, most of the barley grain’s phenolic acids are retained in the husk and become incorporated into the cell wall matrix [43,44]. Phenolic acids have gained increasing attention due to their significant biological activities, including anti-inflammatory, antiatherogenic, antioxidant, and anticancer properties. Owing to its rich nutritional profile and cost-effectiveness, BSG is increasingly being fractioned into distinct components suitable for diverse applications [42].

### Comparative Analysis with Other Grains

A comparison of BSG’s composition with that of unprocessed common cereals and legumes, including unmalted barley, is presented in Table 2.

A comparative analysis of BSG with common cereals and legumes highlights its dietary fiber and protein composition. The fiber content in BSG (40%) is significantly higher than in cereals such as barley (10%), wheat (10%), Oats (10%) and in legumes such as soybean (25%), pea (20%) and white beans (*Phaseolus vulgaris*) (3.23%), highlighting its potential role in improving gut health and digestion. Additionally, BSG’s protein content (30%) surpasses that of barley (15%), wheat (15%) and oats (25%). Although BSG contains less protein than soybean (45%), its protein content surpasses that of peas (27%) and white beans (*Phaseolus vulgaris*) (26.18%), highlighting its rich plant-based protein as a source for food fortification. Unlike most grains, BSG has a lower carbohydrate content (10%), primarily due to the extraction of fermentable sugars during brewing, resulting in a nutrient-dense byproduct that retains valuable proteins and fibers [11,46]. BSG contains a fat content of 7–10%, which is higher than that of most cereals, with essential fatty acids making up the majority of its lipid composition [11,41]. BSG represents a sustainable alternative to conventional grain-based ingredients, offering potential contributions to the circular bioeconomy due to its functional and bioactive constituents. However, comprehensive life cycle assessment (LCA) studies are necessary to fully evaluate and validate the environmental sustainability of utilizing BSG as an alternative nutritional source.

## 3. Functional and Bioactive Constituents of BSG

### 3.1. Dietary Fiber

Arabinoxylan, (1–3,1–4)-β-glucans, and other dietary fibers were officially recognized in 2012 and included in the European Food Safety Authority’s (EFSA) approved list, and they can now be claimed to have health advantages under specific circumstances. The health-related interest in BSG primarily stems from its fiber components, including arabinoxylan and β-glucans, as well as its phenolic compounds like hydroxycinnamic acids [6]. Due to its health-promoting constituents, BSG is utilized in food formulations for its potential to aid in the management of various health conditions, including constipation, metabolic disorders like obesity and diabetes, as well as cardiovascular diseases [1]. Its high dietary fiber content supports cholesterol and fat excretion and may alleviate symptoms associated with ulcerative colitis [3,47,48]. Dietary fibers delay gastric emptying, which prolongs the feeling of fullness and can contribute to reduced calorie intake and weight management [49,50,51]. The food industry is very interested in extracting more promising dietary fibers because fiber has a proven positive impact when included in a healthy diet.

Arabinoxylan (AX), a major component of BSG’s hemicellulose fraction, can constitute up to 25% of its dry weight. This is considerably higher than its presence in barley (4–8%) and wheat (4–10%) [52]. AX consists of a xylan backbone variably substituted with arabinose side chains. Phenolic compounds like ferulic acid can be esterified to these arabinose units. The solubility of AX is influenced by factors such as the arabinose-to-xylose ratio, the degree and pattern of substitution along the xylan chain, and the potential of ferulic acid residues to engage in oxidative cross-linking [6,52]. The solubility of AX is a key factor contributing to its physiological benefits. AX is classified among the indigestible carbohydrates, or dietary fibers, that pass through the digestive system intact and reach the large intestine without structural modification [52]. About one-third of AX is soluble in water, whereas the remaining portion is insoluble [52,53]. A notable fraction of water-soluble AX that reaches the colon serves a prebiotic fiber, undergoing fermentation by beneficial gut microbes such as *Bifidobacterium* and *Lactobacillus* species [6,54,55]. These microorganisms are considered essential for maintaining gastrointestinal health. Additionally, consumption of AX has been associated with improved regulation of postprandial blood glucose levels. AX may help attenuate the postprandial increase in blood glucose through enhancing bulk viscosity, which would postpone stomach emptying and decrease intestinal motility. To produce the desired effect, an intake of 8 g of AX-enriched fiber per 100 g of available carbohydrates is advisable [6,52,56].

Lignin contributes to a significant proportion (11–15%) of BSG. While lignin itself resists digestion in the human gastrointestinal tract, its interaction with the gut microbiota is gaining increasing attention, especially in the context of dietary fiber, polyphenol metabolism, and colonic fermentation. For example, emerging research indicates that the gut microbiota can partially degrade lignin and metabolize the released monomers. An investigation using an in vitro colonic model showed that the lignin-rich fraction enabled the prolonged survival of bifidobacteria more effectively than glucose as a fermentation substrate [57]. Furthermore, phenolic compounds present in BSG have shown a potential DNA protective effect in cell models of oxidative stress [58]. The findings indicated that phenolic-rich extracts of BSG exhibited the strongest defense against H_2_O_2_-induced DNA damage as measured using the Comet assay [58,59]. Among the phenolic acids present in BSG, ferulic acid and *p*-coumaric acid are the most prevalent, occurring at concentrations between 10.6 to 1144 µg/g and 453 to 686 µg/g, respectively. Catechin and vanillin follow as the next most abundant phenolic constituents, as shown in Table 1. Snack bars, chocolate beverages, and yogurt were enriched with phenolic compounds and protein hydrolysates derived from BSG, and their biological activities were assessed through in vitro gastrointestinal digestion models [10]. The resulting digests exhibited protective effects against hydrogen peroxide-induced DNA damage in Caco-2 cells and demonstrated potential immunomodulatory properties [10]. Ferulic acid has been isolated from BSG due to its numerous beneficial health effects, including its ability to reduce allergic responses, modulate inflammation, and suppress microbial activity. Also, *p*-coumaric acid serves as a chemo-protector and antioxidant [52,60]. Furthermore, phenolic acids show promising potential for use in the cosmetic and pharmaceutical sectors due to their bioactive properties [61]. The utilization of phenolic acids in food products promotes the use of this byproduct, which provides the food industry with an innovative food ingredient.

### 3.2. Assessment of the Potential Health Benefits of BSG Using Human Clinical Trials

Emerging research has highlighted the potential of BSG to exert beneficial effects on human health through its provision of dietary fiber, protein, and bioactive compounds with functional properties. Beyond its compositional profile, BSG is of interest for its possible roles in modulating cardiovascular risk factors, supporting metabolic health, and contributing to adequate protein nutrition. However, translating compositional advantages into measurable physiological outcomes requires evidence from well-designed human intervention studies. To date, only a limited number of clinical trials have directly evaluated the effects of BSG consumption on humans, offering preliminary insights into its tolerability, metabolic impact, and capacity to supply essential nutrients. Ummels et al. [62] investigated the postprandial amino acid (AA) uptake kinetics of a protein shake containing barley–rice protein (BRP) isolate derived from BSG in comparison with pea protein isolate (PP) in healthy adults. Each protein shake contained 20 g of protein. In this randomized, double-blind, crossover trial, total amino acid (TAA) uptake from BRP was 69% when compared to that of 87% from BP [62]. The peak height for amino acid concentration was comparable across all protein shakes, indicating similar digestion and absorption rates. Furthermore, the findings indicated that daily consumption of 20 g BSG–derived BRP was well tolerated in healthy individuals, with minimal gastrointestinal symptoms and no significant differences in digestive comfort compared to whey or pea protein shakes. Despite an equivalent carbohydrate composition across treatments, BRP elicited a slightly lower peak insulin response, while glucose responses were comparable to pea protein, suggesting possible differences in carbohydrate type and protein–insulin dynamics [62]. Additionally, the insulinotropic effect of amino acids released during protein digestion, particularly branched-chain AA such as leucine, with variations in composition among proteins, influences the magnitude of the insulin response. The results support BRP as a nutritionally relevant and digestively acceptable plant-based protein source, with postprandial AA appearance patterns that could contribute to essential amino acid supply and muscle protein synthesis stimulation. Further research using isotopic methods and longer-term interventions is needed to confirm these findings and fully quantify digestibility and bioavailability in diverse populations.

A pilot randomized controlled trial by Schmidt-Combest et al. [63] examined the effects of an eight-week intervention with BSG-enriched muffins (8.3 g/day) on cardiovascular disease (CVD) risk markers in healthy adults. The intervention significantly in-creased dietary fiber intake by approximately 5 g/day compared to controls, confirming that BSG can be feasibly incorporated into a habitual diet. It did not significantly impact CVD risk factors in this sample of healthy adults [63]. While no statistically significant changes were observed in lipid profile, fasting glucose, insulin sensitivity, blood pressure, inflammatory markers, or body composition, favorable trends emerged, such as the maintenance of low-density lipoprotein cholesterol (LDL-C) and a modest increase in high-density lipoprotein cholesterol in the BSG group [63]. The absence of gastrointestinal discomfort further supported the tolerability of daily BSG consumption. The results suggest that daily consumption of BSG in the form and dosage used in the study is safe and well-tolerated, but it did not produce significant improvements in CVD risk markers in healthy adults. The absence of measurable effects is likely due to several factors, including the low soluble fiber content of BSG (as most is lost during brewing), the modest daily fiber intake achieved, the relatively small sample size, short intervention period, and the fact that participants were healthy, normocholesterolemic individuals with limited room for improvement in lipid or metabolic biomarkers. Research carried out on whole grains indicates that greater reductions in LDL-C and total cholesterol are generally linked to higher soluble fiber and β-glucan intake [64]. In this study, differences in physical activity levels, dietary macronutrient distribution, and habitual whole grain consumption may also have attenuated any potential effects.

Collectively, these clinical trials provide early evidence that BSG can be safely integrated into human diets, enhance fiber intake, and deliver a postprandial amino acid response comparable to conventional plant proteins. While the cardiovascular biomarker trial did not demonstrate statistically significant improvements in healthy adults, both studies underscore the feasibility, tolerability, and nutritional relevance of BSG-derived products. Further large-scale, longer-term interventions, particularly in populations with elevated disease risk, are necessary to confirm these potential health benefits and to refine optimal dosing strategies.

### 3.3. Contaminants of BSG, Potential Risks, Mitigation Strategies, and Regulatory Reference

BSG can be susceptible to contamination by a variety of naturally occurring and synthetic toxic compounds, posing potential safety risks when used in food or feed applications. However, research to date has examined a limited subset of these compounds in BSG and other agricultural feed materials. Among natural contaminants, mycotoxins represent a primary concern. These toxic secondary metabolites are produced by fungi that readily colonize barley and other cereals used in brewing, both pre- and post-harvest, particularly during on-farm storage under suboptimal environmental conditions [65,66]. While hundreds of fungal toxins have been identified, research in BSG and related feedstuffs has largely focused on a limited group of regulated mycotoxins, including aflatoxins (AFs), fumonisins (FBs), trichothecenes such as deoxynivalenol (DON) and T-2/HT-2 toxins, ochratoxin A (OTA), zearalenone (ZEN), and ergot alkaloids [6,67,68]. Barley, the predominant cereal in beer production, is particularly vulnerable to contamination by mycotoxin-producing genera such as *Fusarium*, *Alternaria*, *Aspergillus*, and *Penicillium*, and the occurrence and concentration of these toxins depend on fungal prevalence, geographic origin, and environmental factors such as temperature and humidity [69]. The contamination of food by mycotoxins is a problem worldwide, which results in economic losses and health concerns.

Artificial contaminants, particularly pesticide residues from conventional farming practices, represent another risk factor. Pesticides are applied to barley to control pests, weeds, and pathogens, thereby improving yield; however, residues can persist in crops, accumulate in the environment, and potentially affect human health. While typically present at lower concentrations than mycotoxins, pesticide residues may contribute to cumulative exposure to mixtures of toxicants and endocrine-disrupting chemicals (EDCs) with implications for food safety and environmental health [70,71]. There is a worldwide guidance value (GV) regulated by the European Commission (EC 2006) [72] for mycotoxins in feed, which include DON, ZEA, and OTA. However, with the current focus on regulated compounds, emerging mycotoxins and less-studied contaminants remain under-investigated in BSG. Given the nutritional potential of BSG, comprehensive contaminant monitoring, including both regulated and emerging toxins, is essential for ensuring its safe incorporation into human food. Analysis of BSG samples revealed the presence of regulated mycotoxins, predominantly zearalenone (ZEN), with a maximum concentration of 32.3 µg/kg, whereas T-2 and HT-2 toxins were detected, showing concentrations of 3.8 and 4.25 µg/kg, respectively [66]. These concentrations are lower than the regulated limits because EC maximum limits for these mycotoxins in feed materials are considerably higher (250–500 µg/kg) and this is due to good quality barley input, effective control of fungal contamination during cultivation and storage, and the partial loss of mycotoxins during brewing, as some toxins partition into the wort rather than the spent grain. DON and OTA were not found in BSG.

The prevention of microbial and mycotoxin contamination in food and feed systems requires an integrated, multi-stage approach encompassing pre-harvest, harvest, and post-harvest phases. Preventive approaches might help to reduce the level of mycotoxin in food but are not sufficient to eradicate mycotoxins. Fresh BSG and other susceptible matrices, contamination risk is strongly influenced by environmental parameters such as moisture content, water activity, and temperature, as well as by the physical integrity and nutrient composition of the substrate [73,74]. Post-harvest management plays a vital role in preventing the growth of toxigenic fungi and spoilage microorganisms. In cereals, rapid drying to a moisture content of 13–14% is effective in inhibiting fungal development, and BSG, with its high moisture and nutrient content, is particularly susceptible to microbial proliferation. To stabilize BSG, refrigeration at or below 4 °C can significantly slow microbial growth as well as acidification. Furthermore, modified atmosphere storage, using low oxygen or elevated CO_2_, reduces aerobic microbial metabolism, and additional interventions such as dehulling, cleaning, and sorting can remove contaminated fractions, while mycotoxin-binding agents or detoxifying enzymes may further mitigate residual contamination risks [73]. Globally, contamination control is supported by a framework of regulatory bodies and guidelines. The Codex Alimentarius Commission, jointly managed by the Food and Agriculture Organization (FAO) and the World Health Organization (WHO), establishes international standards for maximum permissible mycotoxin levels in food and feed, promoting safe food production. Regionally, the European Food Safety Authority (EFSA) provides scientific risk assessments and underpins legislation within the European Union that sets maximum limits. Together, these regulatory frameworks provide quantitative benchmarks for contamination control, ensure routine monitoring and enforcement, and encourage the adoption of preventive measures throughout the production chain.

## 4. Functional Integration of BSG in Food Products

**Figure 1:**
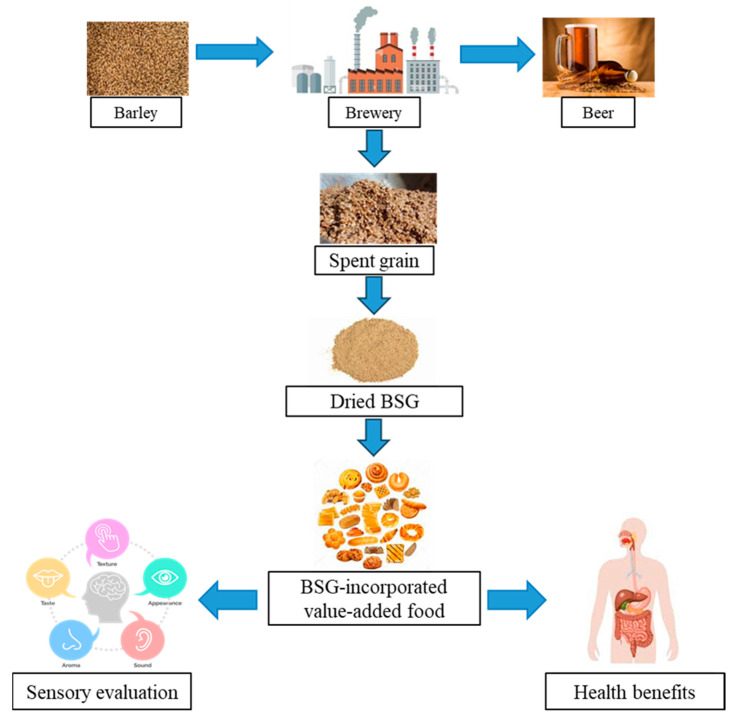
A schematic diagram of BSG production and its uses in value-added food products.

About 100–130 kg of BSG with a moisture content of 70–80% is produced from every 100 kg of malt, yielding around 21–22 kg of BSG per hectoliter of beer brewed [6]. This high moisture content presents two major challenges. Firstly, the cost of transporting wet BSG is relatively high, which is one reason it is traditionally distributed to local farmers for use as animal feed; however, production volumes often exceed local demand [75]. Secondly, due to its elevated moisture level, abundant polysaccharide and protein content are susceptible to microbial development and deterioration, BSG has been recognized as an intriguing area that may hinder its successful use. Although BSG is generally regarded as microbiologically stable and within acceptable safety limits for food applications, the presence and growth of microaerophilic and anaerobic bacteria suggest that its microbial community can shift rapidly after production [76,77]. Therefore, to ensure its suitability for later use, BSG must undergo stabilization and be maintained under optimal post-processing storage conditions. Reducing the moisture content to about 10% is advised to increase storage time (Figure 1). Drying is one of the most prevalent techniques for preserving BSG. In many breweries, BSG is processed using a two-stage drying approach—initially reducing moisture content to below 60% through mechanical pressing, followed by thermal drying to achieve a final moisture level of less than 10% [6,78]. Industrial scale drying remains the most effective preservation technique for BSG. Traditionally, this process has relied on direct rotary drum dryers as the primary equipment [77].

Preservation of BSG through freeze-drying, solar-drying, or oven drying reduces product volume, thereby lowering transportation and storage costs; however, these methods may also influence the composition of its bioactive and nutritional components [78,79,80]. From an economic standpoint, oven drying is more cost-effective than freeze-drying [77,79]. Freezing is not considered an ideal preservation method for BSG, as it can alter or degrade certain sugar (such as arabinose) components of BSG, and oven drying at high temperature increases the risk, potentially leading to a burning effect [80]. Therefore, to protect against unpleasant flavors, BSG must be dried in the oven at temperatures less than 60 °C. Also, the use of sun for drying has been shown to have minimal effect on BSG when exposed for 15 h and stored for almost 180 days, with no significant changes observed in its nutritional or microbiological properties. Following drying, BSG is often subjected to milling to produce a form more appropriate for incorporation into food products (Table 3). This process reduces particle size, improving texture for better consumer acceptance since the untreated material is typically too coarse, and simplifies subsequent analytical procedures [75]. Research has shown the use of the sieving method after milling to separate uneven fractions and to separate them into fine, medium, and coarse fractions for flour production.

### 4.1. BSG as a Functional Food Ingredient in Baked Food

#### 4.1.1. Bread

Bread is a popular food, and the integration of BSG into bread production affects the entire production process, from ingredient mixing to the final product [14]. During mixing, hydration is promoted as ingredients are uniformly combined and interact with water. However, the high fiber content of BSG, particularly due to its abundance of hydroxyl groups, leads to increased water absorption. Consequently, this reduces the free water available during dough development, thereby impairing the formation of the gluten network and the gelatinization of starch [41,102,103,104,105,106]. The addition of spent grain, limited to 10–15%, is recommended because it affects its sensory properties. A higher amount can lead to decreased volume, aroma, and taste changes, as well as alterations in rheological characteristics [83]. Incorporating this byproduct in large proportions can alter the rheological behavior of dough, leading to heightened resistance during biaxial stretching and an increase in the strain-hardening response. This can also lead to bread reduced loaf volume and a denser crumb structure. Conversely, adding spent grain can increase protein content and fiber content [103]. Research conducted using 15% spent grain and 15% sourdough to make bread showed a high fiber with 11.9% fiber content in bread enriched with spent grain flour and 12.1% when spent grain was incorporated through sourdough [107]. The arabinan-to-xylan ratio of approximately 0.45 in spent grain, which is considerably lower than the ratios found in wheat bran (0.88) and wheat endosperm (0.67), causes the bread’s specific volume to decrease when more spent grain is added [1].

In contrast, excessive incorporation of BSG at 20% in bread formulations has been shown to reduce dough development time (DDT), whereas the longest DDT was observed with a 15% BSG inclusion [14,82]. An opposing trend was observed in another study, where BSG addition increased the dough development time while simultaneously reducing dough stability [102,105,106]. The incorporation of spent grain extended the bread’s shelf life by approximately 24 to 48 h, effectively delaying the onset of rope spoilage [1]. However, substituting wheat flour with spent grain adversely affected gluten formation, resulting in lower sedimentation values, decreased dough stability, and increased softening [83]. These effects were largely attributed to extended mixing durations, high shear forces, and the elevated fiber and protein contents of the spent grain [48,81]. The adverse effects of BSG on gluten formation are consistent with established cereal chemistry principles, where dilution of gluten proteins and interference from insoluble fiber have been repeatedly shown to weaken dough rheology. Other high-fiber wheat replacements, such as oat or pulse flours, demonstrate similar reductions in sedimentation value and dough stability, confirming that the mechanical and hydration challenges observed with BSG are not unique but inherent to fiber-rich, non-gluten ingredients. Furthermore, the necessity for longer mixing times with BSG-containing doughs increases the risk of mechanical overdevelopment, which can further degrade the gluten network. At a 10% inclusion level, BSG-incorporated bread maintained similar appearance, crust, and crumb characteristics to control wheat bread (*p* ≤ 0.05), though an increase in BSG content to 20% led to a progressive change in crumb color from light cream to brown [83]. For the purpose of making bread, both untreated and enzyme-treated wasted grain was employed [102]. While the direct addition of spent grain, along with enzymes such as Pentopan Mono BG and Celluclast BG, to the dough improved bread texture and volume, pre-treating spent grain with these enzymes did not significantly influence the final bread quality. There are several benefits of increasing fiber to bread, such as longer DDT, increased dough stability, and crumb firmness but it also has certain drawbacks, such as reduced loaf volume and softening [1]. When high-fiber ingredients such as BSG are added, they dilute the concentration of gluten-forming proteins (gliadin and glutenin) in the flour. This means there’s less protein available to form the continuous viscoelastic network that traps CO_2_ during fermentation and fiber also competes with proteins for water, reducing the hydration needed for optimal gluten development. All of this weakens the dough’s gas-holding capacity, so less expansion occurs, leading to reduced loaf volume and a more coarsed texture. Adding Pentopan Mono BG, Lipopan Extra and a blend of Pentopan Mono BG with Celluclast led to thicker cell walls and lower cell, promoting a more open dough network, thereby improving the bread’s texture, volume, and shelf life [10].

#### 4.1.2. Breadsticks

The incorporation of BSG into baked snacks such as breadsticks markedly enhanced their dietary fiber content, reaching up to 15% [84]. However, this enrichment also altered several baking characteristics. Breadsticks formulated with flour blends containing over 15% of BSG exhibited darker coloration, reduced crispiness, and decreased baking volume, effects attributed to the elevated fiber content introduced by BSG [83,84]. Their shelf life was tracked, and it was discovered that the products’ quality remained in their original state during storage for about 50 days. BSG may be utilized as a functional component in baked foods; however, further study is needed to determine the optimum % of BSG incorporation to retain consumer acceptability by consumers without making a significant impact on the quality of breadsticks.

#### 4.1.3. Cookies and Shortbread

Considering cookies are ready-to-eat food, containing different ingredients and proportions, serve as a convenient energy source, possess an extended shelf life, and are widely consumed across all age groups, making them suitable vehicles for the addition of diverse types of flour. Incorporating 20% BSG into cookies led to reduced starch hydrolysis, a lower glycemic index, and decreased total starch content compared to the control formulation [88]. The high amount of BSG in cookie production caused an increase in fat levels. In cookie making, the substitution of BSG in cookie formulations influenced their physical properties, resulting in increased thickness and width, while a decline in spread ratio was observed in comparison with control samples [85,88]. This is dependent on the amount of BSG added. The use of BSG also reduced the bulk density and water absorption in cookie production and increased the emulsion, DS, DDT, and oil absorption capacities [87,88]. The impact of incorporating fresh BSG (both milled and unmilled) into cookie formulations was assessed. Results indicated that fresh BSG did not compromise the microbiological stability of the final product. In contrast to other baked food products, incorporating BSG up to 30% in cookies did not negatively impact the dough development time and dough stability [1]. Although fresh BSG is generally prone to microbial and chemical degradation due to its composition, the study confirmed the absence of microbial proliferation, including yeasts, molds, *Escherichia coli*, and *Clostridium* species [1,86]. When wheat flour was replaced with about 30% BSG to make shortbread, it resulted in a notable enhancement in both fiber and protein content, maintaining acceptable sensory characteristics but a reduction in carbohydrate content and overall energy value relative to the control formulations [1,89].

#### 4.1.4. Muffins

Introducing BSG into baked formulations adversely affects the spread ratio, primarily due to the increased viscosity of the dough [14]. Nonetheless, because of mechanical factors that occur during mixing and enable the development of a protein network, baking time can increase the spread ratio [108]. The effects of two drying methods, impingement drying and hot air drying, on BSG composition and the quality of muffins incorporated with BSG were evaluated [90]. Replacing 15% of wheat flour with BSG flour in the muffin formulation led to a 23% increase in protein and a 13% rise in total dietary fiber. This substitution did not negatively impact consumer acceptance. The viscosity of the batter increased as a high amount of BSG was added to the muffin recipe. This may be because of the high fiber content in BSG that acts as a thickener by absorbing water in the batter. Enzymatically modified BSG was used in muffin production, and the rheological, textural and sensory properties were compared to muffins with unmodified BSG. Low-level substitution with enzymatically modified BSG was found to lower batter viscosity and muffin hardness while maintaining sensory qualities comparable to muffins containing unmodified BSG [91]. Enzymatic modification of BSG partially breaks down its fiber structure, particularly insoluble polysaccharides, reducing their water-binding capacity and particle rigidity. This structural alteration decreases batter thickening, allowing for lower viscosity and softer muffin texture, while still retaining the nutritional benefits of added protein and dietary fiber. Because the modification reduces the coarse, fibrous mouthfeel often associated with unmodified BSG, it preserves sensory qualities, ensuring consumer acceptance remains comparable. A study found that 30% BSG in muffins retained consumer acceptance while eliciting beneficial biological responses, attributed to elevated protein, fiber, and antioxidant contents [1,109].

#### 4.1.5. Wafers

Wafers are produced in various forms by baking a fluid batter typically composed of wheat flour, water, salt, leavening agents, and flavor-enhancing additives. They are commonly presented as sheet-like structures or with porous, alveolar textures and generally lack filling [1,110]. The drawback is that the products, especially when produced without fillings, tend to have low nutritional density because they are often made from refined flour with minimal protein, fiber, or micronutrients. Additionally, their open texture and simple composition can lead to variability in sensory attributes such as crispness, flavor intensity, and mouthfeel, making it harder to achieve consistent product quality across batches. With better textural properties, wafers strengthened with BSG demonstrated great general appeal; nevertheless, they displayed a darker coloration and reduced fracturability [93]. The inclusion of BSG in wafer production was found to enhance textural parameters such as gumminess, chewiness, springiness, firmness, and cohesiveness, while leading to a decline in adhesiveness [93]. Since water holding capacity and water activity directly influence the crispiness of baked wafers, it is suggested to maintain water activity within the range of 0.387 to 0.52 to preserve this desirable texture [92,111]. However, other factors such as flour type and composition, leavening agents, and baking parameters can influence the crispiness of baked wafers. Analyzing the microstructure of the produced wafers is essential for evaluating product quality. This involves assessing pore size and cell wall thickness across the cross-section. The findings indicate a non-uniform pore distribution, where the central region contains larger pores, while the edges are characterized by smaller pores and denser outer layers [112]. More studies can be done to improve the color, appearance and texture of wafers as well as the shelf life.

#### 4.1.6. Snacks

A crispy snack sample containing 10% of BSG was produced, and the results showed high crispiness index (degree of crispiness) alongside low crispiness work (the amount of mechanical energy (usually in mJ or N·mm) required to initiate and propagate the fracture of a food product), suggesting that the inclusion of this amount of BSG did not adversely impact the crispiness of the final product [112]. Adding a higher quantity of spent grain modified the texture and crumb structure and altered the odor profile. An increase in total polyphenols, protein, fat, dietary fiber, flavonoids, and energy content was observed in the BSG-fortified snack [33]. A study investigating the influence of BSG incorporation and screw speed on the physical and nutritional characteristics of extruded snacks revealed that including up to 30% BSG significantly elevated phytic acid and resistant starch levels [94]. The screw speed did not show a significant impact on total phenolic content or antioxidant activity. In a related experiment examining the effect of BSG on processing parameters, the inclusion of 10% BSG was found to increase the hardness of the product and slightly enhance its expansion [113]. Expansion was enhanced by higher screw speeds and lower moisture levels. Food industries are presently producing new snack bars supplemented with macro- and micronutrients as well as BSG, which promotes acceptability in the snack business.

#### 4.1.7. Pasta

To manufacture pasta, a lot of researchers have investigated using ingredients from agro-industrial byproducts in place of some of the flour [93,95,114,115,116]. Pasta’s great digestibility, delayed release of carbohydrates, low glycemic index in comparison to pizza, bread, and other cereal products, long shelf life, variety, and ease of preparation are the reasons behind its rising global consumption [1,117]. BSG significantly influences pasta production, affecting both pre- and post-cooking properties [95]. Its inclusion compromises protein functionality and leads to increased solid loss during cooking due to excessive starch granule swelling. This hinders the pasta’s ability to recover from rolling deformation and results in reduced elasticity [95]. Pasta enriched with BSG showed elevated levels of protein, fiber, and β-glucans, along with modest improvements in antioxidant activity and acceptable sensory attributes [96]. In another study, a blend of semolina and spent grain was used to produce spent grain-enriched pasta, and it was characterized by high fiber and antioxidant content [96]. It showed that the total antioxidant capacity in pasta rose proportionally with the amount of BSG incorporated. Various attributes of the pasta were assessed, including its proximate composition, textural properties, optimal cooking duration, sensory characteristics, and color profile. An increase in the quantity of spent grain in the pasta caused a change in the color, making it look darker compared to the whole grain semolina pasta sample [96,99,118].

Cooking loss (CL) is a critical indicator of pasta quality, with lower cooking loss (CL) being preferred [1]. Pasta formulations enriched with dietary fiber exhibited reduced CL values (3.47), and an increase in BSG content further contributed to the reduction in cooking loss [97]. Starch gelatinization and protein coagulation are key processes that determine the structural integrity and overall quality of pasta during cooking. The incorporation of BSG-derived ingredients was found to reduce starch gelatinization, primarily due to their elevated protein content [119]. Starch is trapped within a protein network via ionic, hydrogen, and covalent bonds, which limit gelatinization and improve resistance to shear and heat. Higher quantities of starch and protein result in competition for available water, thereby limiting starch swelling during gelatinization. Consequently, the decreased starch content impacts the extent of gelatinization and contributes to reduced cooking loss in pasta. The optimal cooking time of pasta decreased following fortification with BSG, and this could be because of the elevated dietary fiber content. This fiber modification alters the pasta’s structure, facilitating earlier starch gelatinization and enhancing water absorption [120]. Water absorption (WA) during optimal cooking, which reflects starch swelling and gelatinization, is a critical quality parameter for pasta. High-quality pasta typically exhibits a WA range of 150 to 200 g of water per 100 g of pasta [1]. The swelling index (SI) provides information about the integrity of the protein matrix, which restricts water penetration [119,121]. The SI reflects how much a material such as a cereal grain can absorb water and expand. When the protein matrix is intact and well-structured, its dense network of cross-linked proteins and associated cell wall components acts as a physical barrier, restricting water penetration into the interior. This reduced permeability slows hydration, limits starch granule swelling, and maintains the structural integrity of the material. Good quality pasta is characterized by a balanced cooking time, minimal cooking loss, controlled water absorption and swelling, firm texture, low stickiness, and high chewiness [1].

#### 4.1.8. Yoghurt

The texture and sensory characteristics of yogurt are affected by its inclusion of BSG during production. To examine how BSG affects yogurt fermentation, Naibaho [100] blended BSG with milk in different proportions (0, 5, 10, 15, & 20%). The substitute yogurt with 5–10% BSG shortened the fermentation period while maintaining the generation of lactic acid as well as promoting the growth of lactic acid bacteria (LAB). It is noted that a shorter fermentation time accelerates texture formation, resulting in a denser, more viscous product that affects flow behavior [100,122]. The pH increase may help industries use minimal sweetener to offset the acidity and BSG’s high dietary fiber content helped to maintain viscosity at certain addition levels throughout the storage period, reduce syneresis, and increase shear stress [100]. 15–20% BSG reduced the yogurt’s flow performance despite producing the same amount of acidity, LAB and had the least syneresis. Furthermore, BSG enhanced the survival of lactic acid bacteria (LAB) during 14 days of refrigerated storage [100,111]. Similarly, a milk-based yoghurt was fortified with 3 different protein hydrolysates, and its rheological behavior, acidity and LAB were evaluated. The results demonstrated that BSGPs (BSG Proteins) enhanced lactic acid production more effectively than milk protein-based yoghurts. Notably, BSGP-P (BSGP with protease) supported better growth and viability of lactic acid bacteria (LAB), especially improving the survival of *Lactobacillus bulgaricus* [101]. Despite a weak gel formation initially, BSGP-P demonstrated a capacity to create more firm networks, a denser, softer and more homogeneous surface appearance, and maintained consistency of the yogurt throughout storage [101]. This indicates that either blending of BSG or fortifying the protein extract in yogurt promotes lactic acid formation.

### 4.2. Use of BSG in Drinks and Beverages

The creation of functional fruit juices and beverages is being driven by customer desire for the development of functional foods of plant origin that may enhance health and well-being. The antioxidant properties of fruit juices and smoothies enriched with BSG-derived phenolic extracts were evaluated to assess their suitability as functional foods of plant origin. In the analyzed samples, total phenolic content (TPC) varied between 0.37 and 2.15 mg GAE/mL across the fruit juices (grape and cranberry juice) and smoothies (pomegranate and strawberry smoothie) [44]. A maximum inclusion of 10% BSG phenolic extract raised the ferric reducing antioxidant power (FRAP) of cranberry juice, highlighting the potential application of BSG-derived phenolics as natural antioxidants in functional food formulations [1,44]. BSG was effectively utilized to produce a fermented beverage, and it was discovered that it increases dietary fiber, antioxidant content, phenolic components and has a 15-day shelf life for its bioactive constituents [18]. These findings indicate that BSG holds potential as a value-added ingredient in drinks and beverages, offering nutritional enhancements as well as specific sensory attributes.

## 5. Consumer Acceptance of BSG-Incorporated Food Products

Over the past five years, there has been a growing interest in exploring a broader range of food products containing BSG [123]. While earlier applications were primarily restricted to items such as bread and cookies, recent studies have demonstrated the potential for incorporating BSG into a wider array of food matrices. This development reflects the considerable efforts dedicated to valorizing BSG as a functional and health-promoting food ingredient. The highest acceptable inclusion levels of BSG in food formulations are mostly between 10–20% as shown in Table 3. Addition of BSG in lesser amounts yields a positive impact on food acceptability, whereas higher % additions (>20%) decrease the desirability [14]. Consumer studies reveal mixed perceptions regarding BSG-enriched foods. While many consumers recognize its health benefits, concerns about taste, texture, and appearance remain. A hedonic study found that bread with 10% BSG was preferred over higher concentrations [124,125]. BSG addition in extruded products adversely influenced sensory attributes like aroma and aftertaste [126]. This explains the limited levels used in such applications. Similarly, incorporating BSG into cookie production resulted in a noticeable bitter flavor and an overly brittle texture [88]. The panelists were able to tolerate a maximum substitution of 6% [14,85,87] and 10% [14,112] based on the overall acceptability score. It’s interesting to note that panelists still approved of cookies with 20% BSG added. Similarly, the addition of other ingredients such as sprouted pigeon pea and unripe plantain to crackers has increased the consumer acceptability [121,127]. According to processing technology, adding 6.2% BSG to pasta results in highly acceptable pasta [95]. However, panelists believed that a 10% BSG addition during pasta-making was appropriate for its sensory evaluation, which included hedonistic appeal and physical attractiveness [96].

An online study was conducted to assess consumer awareness of BSG using 122 participants from various nationalities. The majority of participants (57.38%) reported no knowledge of BSG prior to the survey, whereas approximately 42.62% were already familiar with it [128]. Interestingly, the participants who knew BSG were those who worked in the education systems, libraries, and students. This suggests that type of occupation has a correlation with the familiarity of BSG and its value, which is important in developing campaigns to educate the population to promote BSG as a sustainable value-added food ingredient. Therefore, when designing public awareness and educational initiatives to introduce or enhance consumer attitudes toward sustainable food choices, demographics should be considered [128,129]. The above study also found that 76.23% of respondents are willing to purchase BSG food products due to their perceived health benefits, contributing to waste reduction, and a sustainable environment. BSG has the potential to be employed in a wide range of food categories, such as pasta, dairy-based products, and baked foods. However, taste, appearance, and pricing are to be customer-friendly to gain consumer acceptance [128,130].

### 5.1. Factors Influencing Consumer Choice

#### 5.1.1. Taste/Aroma

Consumer preference for BSG-enriched bread is predominantly driven by its sensory characteristics, particularly taste and texture [125]. Polyphenol content and composition in BSG have been found to contribute to its bitterness by masking the sweetness of bread and cookie [41]. Discriminatory test was used to analyze the effect of BSG on food product aroma, this revealed that BSG enhanced the aroma [112]. According to reports, BSG contains certain odor components that have a noticeable impact on product aromas, including benzene, 2-heptane, 2-butyl-1-octanol, 3-methyl-butanal, and 2,3-butanedione, and butanal [14,112]. It is noteworthy that the breakdown of proteins, fats, and carbohydrates during fermentation produces odor-active chemicals. Studies have also shown that an increase in BSG for food products has a significant effect on the aroma because a compact texture also prevents the release of odor components [14,112]. The primary distinction between BSG-enhanced bread and wheat bread, according to trained panelists, is the bread’s aroma profile: Panelists’ reactions to a specific aroma and overall aroma impression were elicited by BSG [131]. BSG fermentation resulted in lactic acid and bread with an acidic smell [41]. Consequently, a 10% increase in DSG caused participants to detect an off odor and off flavor in addition to a sour taste and smell [131]. The panelists, however, were unable to specify this influence. Organic acids formed during fermentation may be the source of the acidulous smell [131]. The amount of BSG included in a food product affects the taste which is dependent on the type of food produced. Sensory limitations relevant to BSG could focus on the inherent complexity and variability of BSG as an ingredient. The chemical composition of BSG can vary significantly depending on the type of grain, malting process, and brewing conditions, which introduces inconsistency in flavor and aroma, when incorporated into food products. This variability can make it difficult for panelists or consumers to form consistent sensory impressions, limiting reproducibility and reliability of sensory evaluations. The interaction between BSG and the food matrix, such as moisture retention, crumb structure, or fat content, can also alter mouthfeel, making it challenging to isolate the specific sensory contribution of BSG. Furthermore, panelists’ sensitivity to bitterness, sourness, or off-flavors may vary, meaning that consumer acceptance is highly subjective and context-dependent. Finally, the presence of fermentation-derived compounds may create complex aroma profiles that are difficult to quantify or describe consistently, which limits the ability of sensory panels to fully characterize BSG’s impact.

#### 5.1.2. Color/Appearance

**Figure 2:**
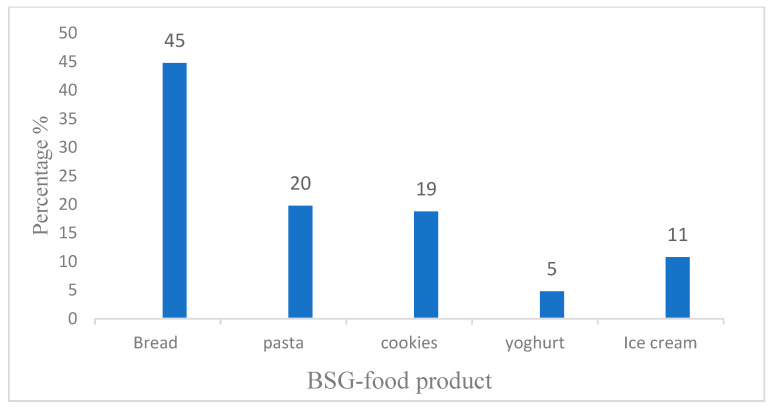
Percentage of consumers’ preference for BSG-added food products based on appearance. Data adopted from [128].

One crucial factor in consumer acceptance of food products is color. Depending on the product, changes of color due to the incorporation of BSG drive the consumer to accept or not accept a new food product [14]. For example, the overall liking score dropped because of the inclusion of BSG, this shows the impact of the color shift after yogurt and cheese production [14,132,133]. Apart from BSG’s propensity for darkness, the Maillard reaction may also contribute to the decrease in brightness [41,108]. Maillard reaction, caramelization, hydrolysis, and pigment degradation are all influenced by temperature [126,134]. Extrusion for breakfast cereal-type products were developed utilizing both fermented and non-fermented BSG. It was observed that because of their appearance, consumers favored the commercial morning cereal. However, a comparison of all the treatments revealed that the 30% fermented BSG was preferred due to its rich flavor, crunchiness and aroma [135]. Additionally, it was discovered that the brightness level was adversely correlated with the hardness of extrusion products [126,134,135] and that a compact texture contributed to the undesirable release of off odor [112]. Therefore, from the standpoint of the consumer, increasing its physical characteristics accomplished by fiber refinement can increase the end products’ acceptability. Based on visual evaluation in research, Bread, pasta, and cookies enriched with BSG were the most favored by participants. As illustrated in Figure 2, bread received the highest preference rating (45%), followed by pasta (20%), cookies (19%), ice cream (11%), and yogurt (5%). These findings highlight the potential of incorporating BSG into food products, particularly in terms of enhancing visual appeal.

The natural dark color of BSG can cause products to appear less familiar or less visually appealing, even if taste and texture are acceptable. This visual bias can influence consumers’ expectations and acceptance, sometimes overriding actual flavor or aroma characteristics. Additionally, color changes resulting from Maillard reactions or thermal processing can vary unpredictably depending on the BSG source and processing conditions, making it difficult to achieve consistent visual quality. The interplay between appearance and texture is another limitation: denser or darker products may also be perceived as harder or less palatable, and compact textures can hinder aroma release, further complicating sensory evaluation. This demonstrates that for BSG-enriched foods, visual perception may act as a limiting factor in overall acceptability, independent of the intrinsic sensory quality of the product.

#### 5.1.3. Texture

The textural properties of products come from mechanical, geometrical, and surface characteristics combined. BSG has been found to increase the hardness of bread, extrusion products, cookies, baked snacks, crackers, biscuits, and cheese blocks [14,41,82,88,103,105,107,126,131,133,135]. Additional attributes assessed were elasticity (bread and pasta), fracturability (extrusion products) and crispness (cookies, crackers, and biscuits) [1,14]. BSG enhances bread hardness by boosting both firmness and springiness, which is due to factors such as arabinoxylans, glucose, and xylo-oligosaccharides in BSG [41,82]. Among various byproducts, BSG exerted a greater influence on the textural properties of bread compared to apple pomace and sugar beet pulp. However, future research is still required for establishing optimized flavor and other characteristics of BSG-enriched bread [136]. The hardness and fracturability of extruded products are influenced by factors such as moisture content, processing temperature, screw speed, and the composition of raw materials [14,126]. BSG increases hardness in two ways: first, by interacting with protein, and second, by trapping water bound in DF after rapid cooling [14,126,134]. Hardness is inversely proportional to the expansion level and fracturability of extrudate products [14,113,126]. In a study of cookie manufacturing, the findings revealed that the hardness decreased marginally with the continued incorporation of BSG due to the brittle texture [88]. The effect was attributed to the brittle texture of high levels of BSG, requiring minimal force to rupture and breaking easily. The addition of BSG disrupts the formation of the network, causing the texture to become more compact and hardens it [14]. These textural changes are largely due to BSG’s fiber and polysaccharide content, which interacts with proteins and binds water, creating a denser product. While in some cases, such as cookies or wafers at high BSG levels, the brittle texture may slightly reduce perceived hardness, the overall effect tends to make products firmer, less elastic, or less aerated than conventional formulations. Depending on the preferred texture, the alteration can mask or interfere with other sensory attributes, limiting the ability of consumers to accurately evaluate the food. Moreover, variations in moisture content, processing conditions, and BSG composition introduce additional inconsistency in texture, making standardization and reproducibility in sensory testing challenging. Consequently, the strong influence of BSG on texture represents a key sensory limitation that must be carefully managed in product development.

## 6. Recommendations and Future Perspectives

To fully leverage the capability of BSG as a sustainable food component, several strategic actions are necessary. Public awareness and education are critical in promoting the adoption of BSG-enhanced food products. Educational campaigns play a crucial role in familiarizing society with food products, thereby changing consumer perceptions and ultimately enhancing their acceptance [128]. Consumers need to be informed about the health benefits and environmental advantages of using BSG, such as its high fiber and protein content, as well as its role in reducing food waste. This can be achieved through targeted campaigns by stakeholders in the food industry, highlighting the sustainability and nutritional value of BSG-incorporated products.

While this review highlights the considerable potential of BSG as a substrate for the development of value-added food products, several critical research gaps remain to be addressed. Future studies should undertake a systematic comparison of BSG derived from different beer types (e.g., ale, lager, and dark beer), as compositional differences may influence functional and nutritional properties. The inherent variability in BSG composition, driven by differences in brewing practices, particularly among craft breweries that do not adhere to standardized technological parameters, should be characterized more explicitly. The potential prebiotic activity of BSG-derived arabinoxylans warrants further investigation, as does the feasibility of incorporating BSG as a protein-rich ingredient in breakfast cereals and granola. Further research is warranted to elucidate the influence of BSG incorporation on the shelf life of value-added food products, to characterize its interactions with packaging materials, and to evaluate its suitability for supporting “clean label” product claims. Additionally, practical challenges associated with large-scale industrial utilization, including effective stabilization and preservation of BSG prior to processing, require rigorous evaluation. Addressing these knowledge gaps will be essential to facilitate the successful commercialization of BSG as a value-added food ingredient.

The optimization of processing techniques is also essential to address challenges associated with BSG, such as its high moisture content and perishability. Innovative and cost-effective drying methods can significantly extend the shelf life of BSG, making it more practical for use in various food applications. Industries must invest in scalable technologies that maintain the nutritional integrity of BSG while reducing storage and transportation costs. Stabilization methods, which prevent microbial spoilage without affecting quality, will further enhance its practicality as an ingredient. Moreover, scaling up these methods through industrial investment can make BSG processing economically viable, ensuring its consistent availability for food manufacturers. Additionally, product development should focus on expanding the variety of BSG-based food items. Baked goods, snacks, functional beverages, and even premium products could incorporate BSG to meet diverse consumer preferences and dietary trends. Another critical aspect is the improvement of sensory qualities in BSG-enriched products. The challenges posed by bitterness, texture changes, and darker coloration should be addressed through advanced processing and formulation techniques. For example, modifying the fiber structure or blending BSG with other ingredients can enhance the flavor, texture, and visual appeal of products, thus improving their overall acceptability [128].

Collaboration between governments, research institutions, and industries is crucial to driving the widespread adoption of BSG. Policymakers can play a vital role by providing financial incentives, grants, and subsidies that encourage the use of agro-industrial byproducts. Techniques to reduce bitterness, optimize texture, and maintain product color and aroma should be prioritized to increase consumer satisfaction and continue to explore the functional properties of BSG, particularly its behavior in different food systems. Regulations that support sustainable practices and reduce barriers to innovation will be instrumental in fostering industrial-scale adoption. These efforts can lead to the discovery of novel applications for BSG, such as its use in biodegradable packaging or providing bioactive compounds for pharmaceuticals. A multi-faceted strategy combining consumer education, technological advancements, collaborative policies, and continuous scientific exploration is essential to unlock the optimal value of BSG and prevent food contamination from mycotoxins and pesticides. By addressing these areas, the brewing and food industries can transform BSG from industrial residue to a useful resource, aligning with global sustainability goals and meeting the increasing demand for nutritious and eco-friendly food products.

## 7. Conclusions

Industries can help to tackle global food insecurity by turning food waste and byproducts into high-value ingredients. Brewer’s spent grain (BSG) shows strong potential as a functional food ingredient due to its rich content of nutrients like carbohydrates, dietary fiber, proteins, and essential amino acids. Research suggests that adding up to 15% BSG to food products offers nutritional benefits without compromising quality. However, higher amounts may negatively affect texture and appearance. To successfully use BSG in food, innovative processing techniques and collaboration across sectors (agro-industry and health) are essential. With growing consumer interest in sustainable and healthy foods, BSG offers a promising opportunity for innovation in the food sector, aligning with global sustainability goals.

## Figures and Tables

**Table 1:** Nutrients and bioactive phytochemical composition of BSG.

Components	Amount	References
Moisture (% DW)	70–80	[12,13]
Carbohydrate (% DW)	50–60	[14,15]
Sugars (% DW)		
Maltose	5–20	[16,17]
Glucose	2.3–23	[16,17]
Maltotriose	0.7	[17]
Fructose	0.4	[17]
Galactose	0.77	[16]
Xylose	12.96	[16]
Arabinose	6	[16]
Mannose	0.94	[16]
Cellulose (% DW)	15–20	[18,19,20]
Hemicellulose (% DW)	15–30	[20,21,22]
Protein (% DW)	15–30	[13,14,17]
Lipids (% DW)	4–10	[13,23]
Ash (% DW)	2–4	[15,20,24]
Dietary fiber (% DW)	30–60	[13,17,25]
Arabinoxylan (% DW)	10.37	[6]
β-glucans (% DW)	1	[26]
Fatty acids (% DW)	2–19	[27,28]
Fatty acid profile (µg/g DW)		
Palmitic acid	2000–3200	[27,29,30]
Linoleic acid	500–4270	[27,29,30]
Myristic acid	25–50	[27,30]
Pentadecylic acid	10	[30]
Margaric acid	110–250	[27,30]
Oleic acid	100–700	[27,29,30]
Stearic acid	90–650	[27,29,30]
Linolenic	50–500	[29,30]
Arachidic acid	25–300	[27,30]
Behenic acid	250	[27]
Decosadienoic acid (Omega-6)	1085	[30]
Tricosylic acid	25.73	[30]
Essential amino acids (% DW)		
Valine	1–4	[12,31]
Isoleucine	2–10	[12,31]
Leucine	0.4–7	[12,31]
Methionine	0.87–3.7	[12]
Threonine	0.8–4	[12,31]
Lysine	0.5–6	[12,31]
Histidine	1.26–5.9	[12,31]
Tryptophan	1	[31]
Non-essential amino acids (% DW)		
Alanine	1–4	[12,31]
Arginine	1–8	[12,31]
Aspartic acid	2–9	[12,31]
Glutamic acid	5–15	[12,31]
Proline	2–10	[12,31]
Serine	5	[31]
Tyrosine	8	[31]
Glycine	1–4	[31]
Cysteine	0.47	[12]
Minerals (μg/g DW)		
Calcium	1000–3600	[29,32]
Magnesium	1900	[32]
Phosphorus	4000–6000	[29,32]
Sodium	137	[32]
Potassium	1570	[29]
Zinc	67.2	[29]
Manganese	34.3	[29]
Iron	210	[29]
Vitamins (μg/g DW)		
B_1_	25	[33]
B_2_	2–25	[17,33]
B_6_	9	[33]
K	4.5	[33]
Polyphenols (μg/g)		
Vanillin	27.18	[34]
Catechin	29–116.04	[16,34]
*P*-Coumaric	453–686	[16,35]
Ferulic acid	10.56–1144	[16,34]
Caffeic acid	0.28	[35]
4-Hydroxybenzoic	14–41.3	[34,35]
Phytosterols (μg/g DW)		
Campesterol	250	[27]
Stigmasterol	50	[27]
Sitosterol	450	[27]
Δ5-Avenasterol	60	[27]
Δ7-Stigmastenol	20	[27]
Δ7-Avenasterol	14	[27]
24-Methylenecycloartenol	40	[27]
Campestanol (ergostanol)	20	[27]
Sitostanol (stigmastanol)	6	[27]
Lignin (% DW)	11–15	[36,37,38]

The values are expressed as a percentage of dry weight (% DW), or with an appropriate unit of measurement.

**Table 2:** BSG nutrient values compared with other unprocessed legumes/cereals [11,45].

Byproduct		Cereals			Legumes/Pulses		
Nutrient (% DW)	BSG	Barley	Wheat	Oats	Soybean	Pea	White Beans
Carbohydrate	15	60	60	55	20	45	54.79
Fat	10	5	5	8	15	4	4.42
Fiber	40	10	10	10	25	20	3.23
Protein	30	15	15	25	45	27	26.18
Ash	3	5	2	2	5	4	7.18

The values are presented as percentages of dry weight (% DW).

**Table 3:** BSG-incorporated food products and their characteristics.

Food Product	Quantity (%)	Characteristics	References
Bread	10–15%	1. Enhanced nutritional composition 2. Color changes from light cream to brown 3. Increased water absorption capacity with high amount of spent grain 4. Increased crumb firmness 5. High fiber content 6. Rheological and pasting properties of dough were affected 7. Increased shelf life	[1,48,81,82,83]
Bread sticks	15%	1. Reduction in loaf volume 2. Less crispy, darker and decreased baking volume 3. 15% BSG increased dietary fiber 4. Increased shelf life	[6,84]
Cookies	10–20	1. Reduced bulk density and water absorption 2. Dough forming time and dough stability increased 3. Increased emulsion and oil absorption capacity 4. Increase in the protein and fiber content 5. Total antioxidant activity increases	[85,86,87,88]
Short bread	30%	1. Reduced carbohydrate content as well as the energy value. 2. Elevated fiber and protein content	[1,89]
Muffins Wafers	15–30% 5–15%	1. Increased viscosity of the batter increased 2. Reduced muffin hardness 3. Enhances the levels of fat, protein, and total dietary fiber 1. Increase in the gumminess, chewiness, hardness of the product. 2. Increased firmness and cohesiveness. 3. Adhesiveness decreased.	[14,90,91] [92,93]
Snacks	10–15%	1. Increased phytic acid and resistant starch content of the product 2. β-glucan, polyphenols and flavonoids exhibited increased concentrations 3. Decreased cell size, limited product expansion, and lower bulk density	[33,81,94]
Pasta	5–20%	1. The increase of the spent grain affects the color of the pasta. 2. A solid structure with higher firmness 3. Decreased cooking loss. 4. Decreased degree of starch gelatinization. 5. Shortened the optimal cooking duration 6. Enhanced nutritional profile	[1,95,96,97,98,99]
Yoghurt/Yoghurt fortified with BSGP	5–10%	1. Improved quality of yoghurt 2. Increased viscosity and shear stress 3. Shortened fermentation time 4. Improved lactic acid formation 5. Helps in growth and survival of LAB 6. Long shelf life (14 days)	[100,101]
Fruit juice and smoothies/beverages	0–10%	1. Increased antioxidant activity 2. Increased shelf life and phenolic components	[1,18]

## Data Availability

Not applicable.
